# Toxicology of *Gambierdiscus* spp. (Dinophyceae) from Tropical and Temperate Australian Waters

**DOI:** 10.3390/md16010007

**Published:** 2018-01-01

**Authors:** Michaela E. Larsson, Olivier F. Laczka, D. Tim Harwood, Richard J. Lewis, S. W. A. Himaya, Shauna A. Murray, Martina A. Doblin

**Affiliations:** 1 Climate Change Cluster, University of Technology Sydney, P.O. Box 123 Broadway, Sydney, NSW 2007, Australia; laczka.olivier@gmail.com (O.F.L.); shauna.murray@uts.edu.au (S.A.M.); Martina.Doblin@uts.edu.au (M.A.D.); 2 Cawthron Institute, 98 Halifax Street East, Private Bag 2, Nelson 7010, New Zealand; Tim.Harwood@cawthron.org.nz; 3 Institute for Molecular Biosciences, The University of Queensland, Brisbane, QLD 4072, Australia; r.lewis@imb.uq.edu.au (R.J.L.); h.siddhihalu@imb.uq.edu.au (S.W.A.H.)

**Keywords:** Benthic Harmful Algal Bloom (BHAB), Ciguatera Fish Poisoning (CFP), Ciguatoxin (CTX), Fluorescent Imaging Plate Reader (FLIPR), Liquid Chromatography-Tandem Mass Spectrometry (LC-MS/MS), Maitotoxin (MTX)

## Abstract

Ciguatera Fish Poisoning (CFP) is a human illness caused by the consumption of marine fish contaminated with ciguatoxins (CTX) and possibly maitotoxins (MTX), produced by species from the benthic dinoflagellate genus *Gambierdiscus*. Here, we describe the identity and toxicology of *Gambierdiscus* spp. isolated from the tropical and temperate waters of eastern Australia. Based on newly cultured strains, we found that four *Gambierdiscus* species were present at the tropical location, including *G. carpenteri*, *G. lapillus* and two others which were not genetically identical to other currently described species within the genus, and may represent new species. Only *G. carpenteri* was identified from the temperate location. Using LC-MS/MS analysis we did not find any characterized microalgal CTXs (P-CTX-3B, P-CTX-3C, P-CTX-4A and P-CTX-4B) or MTX-1; however, putative maitotoxin-3 (MTX-3) was detected in all species except for the temperate population of *G. carpenteri.* Using the Ca^2+^ influx SH-SY5Y cell Fluorescent Imaging Plate Reader (FLIPR) bioassay we found CTX-like activity in extracts of the unidentified *Gambierdiscus* strains and trace level activity in strains of *G. lapillus.* While no detectable CTX-like activity was observed in tropical or temperate strains of *G. carpenteri*, all species showed strong maitotoxin-like activity. This study, which represents the most comprehensive analyses of the toxicology of *Gambierdiscus* strains isolated from Australia to date, suggests that CFP in this region may be caused by currently undescribed ciguatoxins and maitotoxins.

## 1. Introduction

Ciguatera Fish Poisoning (CFP) is a human illness which arises from the consumption of marine fish contaminated with ciguatoxins (CTX) [1]. Globally, it is estimated that between 50,000 and 200,000 people each year are affected [2], making it the most prevalent nonbacterial human illness associated with seafood consumption [3,4]. The risk of CFP is higher in tropical locations and is particularly prevalent among island communities who rely on seafood for survival [5,6], affecting up to 2000 per 100,000 people each year [7]. However, with the onset of international travel and worldwide seafood trade, this foodborne syndrome has now become a global issue [8,9,10].

Some species of dinoflagellates from the genus *Gambierdiscus* produce CTXs, the causative agents of CFP [11]. Adachi and Fukuyo [12] originally described *Gambierdiscus toxicus*, the first species in the genus, from samples collected in the Gambier Islands, French Polynesia. For many years, the genus was thought to be monotypic; however, increased interest and the application of molecular techniques toward identification procedures has led to its reclassification into at least 15 *Gambierdiscus* species and five sub-groups [13,14,15,16]. Targeted sampling and continued research will likely reveal more diversity in the future. The identification of *Gambierdiscus* species based on thecal plate morphology is very challenging, as the differences between species are subtle and largely overlap [12,17,18,19]. Assessment of the phylogenetic relationships of species based on particular genetic markers constitutes additional information for species identification [13,19]. Phylogenetic analysis of *Gambierdiscus* is primarily inferred from the nuclear-encoded ribosomal RNA gene (rDNA), including multiple regions of the large subunit (LSU D1–D3 and LSU D8–D10) [13,19], the small subunit (SSU) or the internal transcribed spacer (ITS) [13].

CTXs are highly potent, lipophilic, polyether toxins which affect mammalian cells by activating voltage gated sodium channels [20,21,22]. Different structural forms of CTXs can be produced by *Gambierdiscus* species (e.g., P-CTX-3B, P-CTX-3C, P-CTX-4A, P-CTX-4B), each displaying differing toxicities [23]. Furthermore, CTXs isolated from marine fish are different from those produced by *Gambierdiscus* species [24,25] and can differ between locations (e.g., Atlantic Ocean vs Pacific Ocean) [26,27,28,29]. Maitotoxins (MTXs) are also produced by species of *Gambierdiscus* [30]. These large, potent polyether toxins enhance intracellular calcium levels [31,32,33] and are amongst the most lethal non-proteinaceous natural compounds known [34].

Routine monitoring of toxins (particularly CTXs) produced by *Gambierdiscus* species is complicated by the many structural differences in the natural products produced, typically low observable quantities, and the lack of adequate reference standards [1]. Currently, there is, no validated unified approach for evaluating the toxicity of *Gambierdiscus* strains. Traditionally, the most common assessment of *Gambierdiscus* toxicity was via the mouse bioassay [19,23]. This involves administering the extracted toxin intraperitoneally or orally to Swiss albino mice, observing the symptomatology and calculating the median lethal dose (LD_50_) [35]. This method however is non-specific and does not allow identification of the compound responsible for the observed toxicity. More recently, mammalian cell-based assays have been developed which assess the toxicity of a microalgal extract and provide a degree of differentiation amongst toxin classes. These include neuroblastoma (neuro-2a) cytotoxicity [36]; human erythrocyte lysis [37] and the Ca^2+^ influx SH-SY5Y cell Fluorescent Imaging Plate Reader (FLIPR) bioassay [38]. Liquid Chromatography-Tandem Mass Spectrometry (LC-MS/MS) is another analytical technique that provides the most specific evaluation of toxins but is limited by the number of forms for which standards are available. This method can detect microalgal-derived characterized toxins such as P-CTX-3B, P-CTX-3C, P-CTX4A, P-CTX4B, MTX-1 and putative MTX-3 [39,40,41].

There have been large outbreaks of CFP in Australia, with two human fatalities and more than 1400 documented cases between 1965 and 2010 [42,43]. Outbreaks primarily occurred in Queensland [42,44,45] and the Northern Territory [46], with one case from Victoria traced back to the consumption of a Maori wrasse (*Cheilinus undulates*) imported from the Great Barrier Reef [46]. More recently, there have been multiple cases of ciguatera in northern New South Wales, linked with the consumption of locally caught Spanish mackerel (*Scomberomorus commerson*) contaminated with P-CTX-1B [47].

Within Australia, the presence of *Gambierdiscus* has been documented throughout the Great Barrier Reef, Queensland [48,49,50,51,52], at Exmouth in Western Australia [53] and at several sites in temperate locations in New South Wales [54]. However, complete identification of the species and toxicology analyses were not included in these studies. Work to characterize the diversity within Australia is underway, with *G. carpenteri* and a species much like *G. belizeanus* described at several locations in the Great Barrier Reef [55,56], as well as *G. carpenteri* being documented at Merimbula and Wapengo Lagoon, New South Wales [57]. Interestingly, the species of *Gambierdiscus* found in Exmouth, Western Australia was similar to *G. carpenteri* [53], suggesting this species is well adapted to Australian conditions and may be widely distributed. More recently, *G. lapillus* was described from samples collected at Heron Island [58] and a previously unidentified strain of *Gambierdiscus* from the same location [15] was reclassified as a new species, *G. honu* [40]. Although our understanding of the identity of *Gambierdiscus* species in Australia is advancing, very little is known about their toxicology (Table 1).

In this study, single cells of *Gambierdiscus* from tropical and temperate Australia were isolated, and monoclonal cultures established. To verify their identity to species level, DNA was extracted from cultures and the D1–D3 and D8–D10 regions of the large subunit ribosomal RNA gene were sequenced. Toxicity was assessed using LC-MS/MS and the Ca^2+^ influx SH-SY5Y cell FLIPR bioassay, advancing our understanding of the organisms which contribute to CFP in Australia.

## 2. Results

### 2.1. Strain Identification

**Figure 1:**
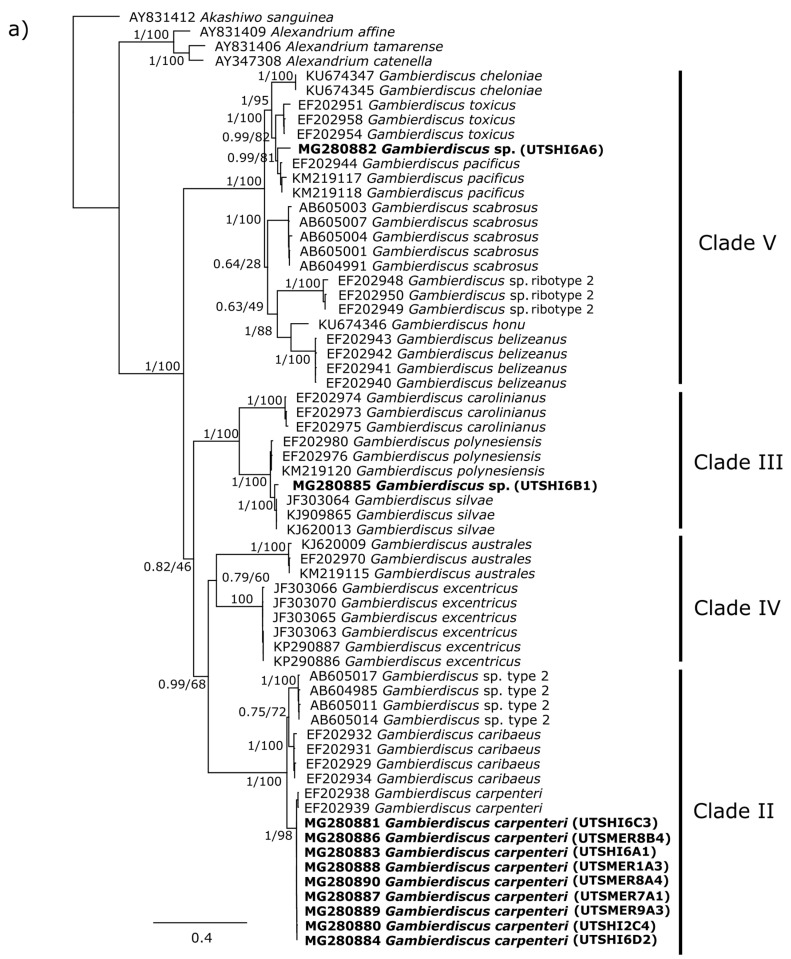

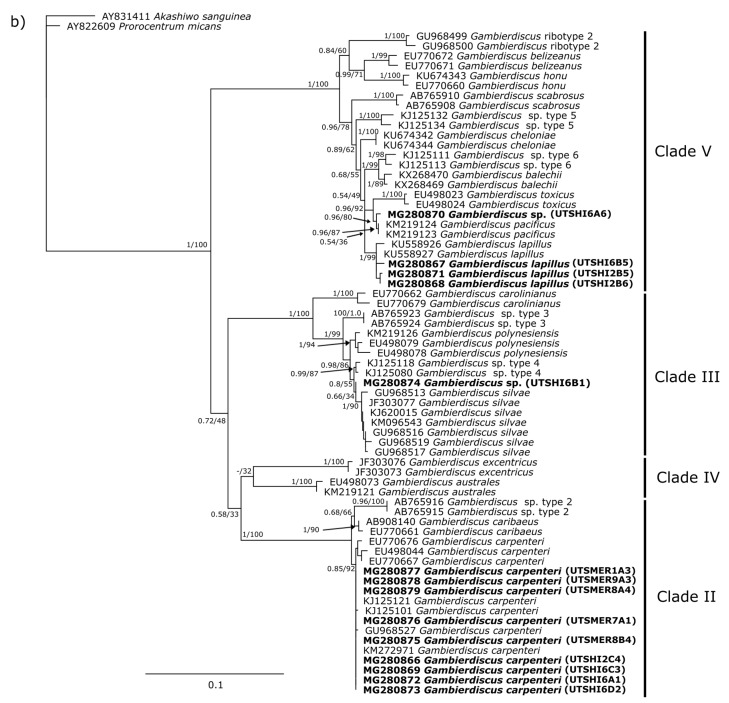
Maximum Likelihood phylogenetic tree showing alignment of the D1–D3 region (**a**) and the D8–D10 region (**b**) of the large subunit (LSU) rDNA sequences. Strains from this study are shown in bold. Values at nodes represent Bayesian posterior probability and Maximum Likelihood bootstrap support.

Maximum Likelihood and Bayesian analyses of the LSU rDNA D1–D3 and D8–D10 regions provided evidence to support the clades described in Nishimura et al. [59] for *Gambierdiscus* species. All five temperate strains of *Gambierdiscus* (UTSMER9A3, UTSMER8B4, UTSMER7A1, UTSMER1A3, UTSMER8A4) and four tropical strains (UTSHI2C4, UTSHI6C3, UTSHI6A1, UTSHI6D2), group with other strains of *G. carpenteri* in both phylogenies with high support (Figure 1a,b). Therefore, most of the isolates collected from eastern Australia were identified as *G. carpenteri* (Table 2). Three strains (UTSHI6B5, UTSHI2B6, UTSHI2B5) group with high support in the LSU rDNA D8–D10 region phylogeny with *G. lapillus* (Figure 1b), a new species described from the same tropical location in eastern Australia (Heron Island) [58]. Sequences were not available however, to compare the LSU rDNA D1–D3 region phylogeny. Strain UTSHI6A6 groups within clade V, the most diverse *Gambierdiscus* clade [59], in both LSU gene phylogenies. This strain is closely related to, but distinct from, *G. pacificus* strains from around the world (Figure 1a,b). Similarly, strain UTSHI6B1 groups within clade III [59] and is closely related to *G. silvae* in both phylogenies, but also forms a distinct cluster (Figure 1a,b). More analyses are required to fully describe these strains and both are therefore referred to as *Gambierdiscus* sp. in this study.

### 2.2. Detection of Characterized Ciguatoxins (CTXs) and Maitotoxins (MTXs)

Analysis of the *Gambierdiscus* culture extracts using LC-MS/MS did not reveal the presence of the microalgal-derived ciguatoxins P-CTX-3B, P-CTX-3C, P-CTX-4A and P-CTX-4B (for which we had calibration standards), or MTX-1, in any of the strains isolated in this study. Putative MTX-3 was detected in all tropical strains of *Gambierdiscus* including *G. carpenteri,* but was absent from all temperate strains of the same species (Table 2). These toxin profiles did not change when the cultures were grown at 18 or 27 °C (Table 2). Tropical strains of *G. carpenteri* produced MTX-3 at temperatures as low as 18 °C and at 27 °C and the temperate cultures did not produce MTX-3 at either temperature.

### 2.3. Presence of Ciguatoxin (CTX) and Maitotoxin-Like (MTX) Activities in Isolated Strains

**Figure 2:**
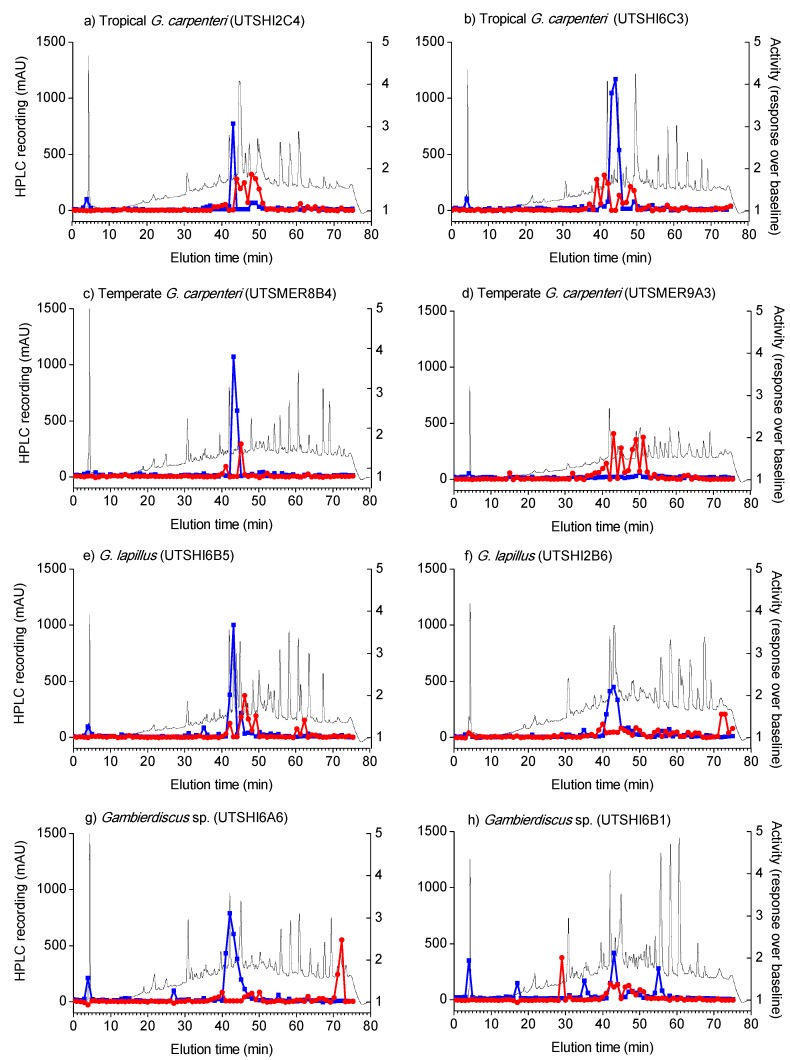
Ciguatoxin-like (red) and maitotoxin-like (blue) activities of the High Performance Liquid Chromatography (HPLC) fractionated (black trace) dichloromethane phase of extracts from *Gambierdiscus* strains isolated in this study.

The Ca^2+^ influx SH-SY5Y cell FLIPR bioassay [38] showed distinct CTX-like activities (i.e., post veratridine addition) in several of the High Performance Liquid Chromatography (HPLC) fractions from the dichloromethane phase of three of the four *Gambierdiscus* species. Two strains, *Gambierdiscus* sp. (UTSHI6A6) and *Gambierdiscus* sp. (UTSHI6B1), showed clear CTX-like activities (Figure 2g,h, Table 2) and low levels of CTX-like activity were also detected in both strains of *G. lapillus* (Figure 2e,f)*.* No distinct CTX-like activity was detected for tropical or temperate strains of *G. carpenteri* (Figure 2a–d). MTX-like activity was detected in fractions eluted at 42–45 min in extracts of all species except UTSMER9A3, one of the temperate strains of *G. carpenteri* (Figure 2d). CTX-like activity was also detected in the fractions eluted prior to, during and following this MTX-like activity peak (Figure 2).

**Figure 3:**
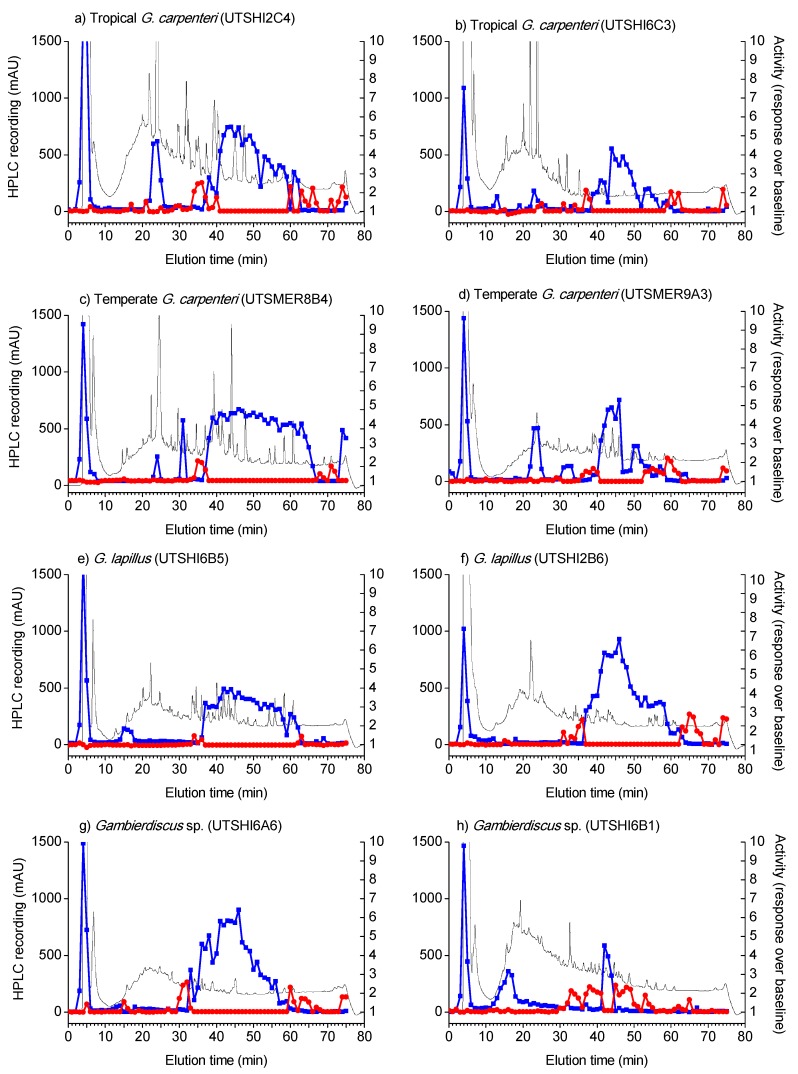
Ciguatoxin-like (red) and maitotoxin-like (blue) activities of the HPLC fractionated (black trace) methanol phase of extracts from *Gambierdiscus* strains isolated in this study.

In comparison to the variable CTX-like activities amongst strains, strong MTX-like activities were detected in all *Gambierdiscus* strains tested. There was a distinct peak in the earliest fractions of the HPLC-fractionated methanol phase and large activity in the fractions eluted between 30 and 60 min for all extracts (Figure 3). All the strains of *G. carpenteri* from both tropical and temperate Australia showed additional MTX-like activity peaks (Table 2) in fractions eluted between 10 and 30 min (Figure 3). CTX-like activity was also detected in most extracts, prior to and following strong MTX-like activity (Figure 3).

## 3. Discussion

CFP has been an issue in Australia for some time but very little is known about the toxicology of the causative organism/s in this region. Prior to this study, strains from various Australian sites had been identified but a thorough analysis of their toxicology had not been undertaken. Identifying the *Gambierdiscus* species and toxins responsible for CFP in Australia is a critical first step toward improving risk management strategies and helping safeguard consumers and the seafood industry. In this study, we established 14 strains of *Gambierdiscus* from eastern Australia, identifying four *Gambierdiscus* species at a tropical site, two of which could represent new species, and one species at a temperate site. Using a functional bioassay approach, we demonstrated that extracts of *G. lapillus* and two other *Gambierdiscus* species (*Gambierdiscus* sp., UTSHI6A6 and UTSHI6B1) show CTX-like activities. Furthermore, based on the LC-MS/MS results, we verified that this activity is not due to CTXs for which we have standards and that are usually tested for in *Gambierdiscus* extracts (P-CTX-3C, P-CTX-3B, P-CTX-4A, P-CTX-4B).

In this study, we uncovered considerable diversity of *Gambierdiscus* at the tropical site, recording four co-occurring species from Heron Island, Queensland. *G. carpenteri* was the only species found to occur at both the tropical and temperate locations (Merimbula, New South Wales). This species is one of the most widely distributed in the genus, occurring in the North Atlantic Ocean [37,60], North Pacific Ocean [13,36,37,38,61,62], South Pacific Ocean [60] and the Caribbean Sea [37,38]. In Australia, *G. carpenteri* has previously been documented in the central Great Barrier Reef in Townsville, Queensland [55,56], and at Merimbula and Wapengo Lagoon, New South Wales [57]. Our results, therefore verify earlier reports of *G. carpenteri* from tropical and temperate Australia.

*Gambierdiscus lapillus* was also identified from the tropical site. It was described by Kretzschmar et al. [58] from the same collection site at Heron Island and is currently the only other record of this species. However, increased sampling and identification of *Gambierdiscus* species will likely reveal a much larger distribution. Two additional strains of *Gambierdiscus* established in this study (*Gambierdiscus* sp.) were not genetically identical to other currently described species within the genus. These may represent additional undescribed species from Heron Island, although further morphological and molecular identifications are required to verify this. A recently described species of *Gambierdiscus* (*G. honu*) [40], has a wide distribution across the South Pacific Ocean, including Heron Island [15], but this species was not isolated in our study. This result highlights that a single sample collection and subsequent isolation process does not necessarily reveal all the diversity of a genus at a specific location. 

The LC-MS/MS analyses did not detect any characterized microalgal CTXs (P-CTX-3C, P-CTX-3B, P-CTX-4A and P-CTX-4B) in any of the culture extracts. However, CTX-like activity was identified in three of the four species tested. Previous studies of the toxicology of *Gambierdiscus* from Australia are consistent with these results (Table 1). Using the same LC-MS/MS method, Kretzschmar et al. [58] did not detect characterized microalgal CTXs in strains of *G. lapillus* isolated from Heron Island but noted the presence of unassigned peaks in the CTX transition zone. Similarly, Kohli et al. [57] did not detect characterized microalgal CTXs in a collection of *G. carpenteri* cells taken directly from the field at Merimbula. The LC-MS/MS method used in these studies was targeted toward the identification of P-CTX-3C, P-CTX-3B, P-CTX-4A and P-CTX-4B only, as these are the currently characterized forms of microalgal origin for which standards are available. Structurally related compounds with different masses are therefore not recognised. This indicates that the CTX-like activity detected using the functional assay in our study, is likely caused by compounds which differ from the currently characterized CTXs of microalgal origin.

*Gambierdiscus* sp. (UTSHI6A6) produced the most distinct CTX-activity peak eluting at approximately 72 min. In a study using the same cell-based functional assay, Lewis et al. [38] identified CTX-like activity which eluted at the same time in strains of *G. ruetzleri* (now *Fukuyoa ruetzleri*), *G. carolinianus* and *G.* ribotype 2. However, LC-MS/MS analyses have not been performed on the strains from Lewis et al. [38], so it is not known whether this activity is linked with known CTX congeners. The results from our study suggest that this may be a novel type of lipophilic toxin with CTX-like activity and its characterization should be the target of future research. Both strains of *G. lapillus* also showed CTX-like activity, although the elution time of the active fractions differed between strains. These differences may arise from strains producing different congeners of a toxin; however, understanding the reasons for such differences require further investigation. 

*Gambierdiscus* sp. (UTSHI6B1) showed distinct CTX-like activity in a low polarity fraction collected at approximately 29 min from the dichloromethane phase where lipophilic compounds like CTXs accumulate. Known ciguatera toxins however, typically have higher polarities, so further research is needed to accurately classify this active fraction. *G. carpenteri* strains from tropical and temperate Australia did not show any distinct CTX-like activities, consistent with Lewis et al. [38] who tested two strains of *G. carpenteri* (originally isolated from Belize in the Caribbean Sea and Hawaii in the eastern North Pacific Ocean), using the same functional cell-based Ca^2+^ influx FLIPR bioassay. These results suggest that *G. carpenteri* does not produce detectable quantities of ciguatera causing sodium channel activation compounds and therefore, may not contribute to the occurrence of CFP.

Many *Gambierdiscus* species display toxicity in the lipophilic phase, following partitioning in assays such as the mouse bioassay and the mammalian cell-based neuroblastoma (N2a) assay, suggesting that CTXs or compounds with the same mode of action are present [16,36]. Efforts should be focused on testing whether the toxicity of these strains can be attributed to the CTXs characterized from French Polynesia, or whether toxins responsible for CFP differ between locations, as our findings seem to suggest. Certainly, our results suggest the CTX-like toxins from Australia may differ from those found in French Polynesia. CTXs isolated from marine fish also differ in structure from the CTXs produced by *Gambierdiscus* species, a result of bioaccumulation and biomagnification; however, the differences in the CTXs isolated from marine fish originating from different locations could suggest that the precursor toxins produced by *Gambierdiscus* sp. are just as diverse.

Large MTX-like activities were identified in the dichloromethane phase of all *Gambierdiscus* cell extracts, except for one strain of *G. carpenteri* isolated from temperate Australia. This was unexpected, as MTXs should only be present in the methanol phase following liquid partitioning of the microalgal cell extracts. However, the elution time of the MTX-like activity peak in the dichloromethane phase corresponds to a fraction with strong MTX-like activity in the methanol phase and therefore, likely represents a carryover of MTXs. Although liquid partitioning of lipophilic (CTX) and hydrophilic (MTX) compounds in cell extracts using dichloromethane and aqueous methanol is effective, some MTX carryover can occur. This was also observed at the same elution time in the original Ca^2+^ influx SH-SY5Y cell FLIPR bioassay method description by Lewis et al. [38]. CTX-like activity was also detected on the shoulders of this MTX-like activity. This is likely a result of sensitization of the sodium channels on the mammalian cell membrane in response to the addition of veratridine, resulting in a CTX-like activity peak and therefore, was not interpreted as an indicator of the presence of potential CTXs in this study. The MTX peak was not detected in *G. carpenteri* strain UTSMER9A3; however, CTX-like activity was. This was likely due to a lower level of MTX carryover from the methanol phase, only high enough to show a response after the addition of veratridine. 

Yasumoto et al. [11] were the first to establish the link between CFP and the dinoflagellate *Gambierdiscus.* The structure of the microalgal derived CTXs responsible were later elucidated from a strain (RG1-1) of *Gambierdiscus* (reported as *G. toxicus* but taxonomic identity is uncertain) isolated from the Gambier Islands in French Polynesia [29,63,64,65,66]. Chinain et al. [23] then went on to complete the first comprehensive characterization of CTXs from two highly toxic strains of *G. polynesiensis* (TB-92 and RG-92) from French Polynesia, and the toxins characterized in these studies remain the primary toxins linked with CFP today.

*G. polynesiensis* has been consistently found to exhibit considerable CTX-like activity (receptor binding assay [23]; mouse bioassay [39]; neuro-2a Assay [67]) and is the only species shown to produce the characterized CTXs when tested using LC-MS/MS analyses [23,39]. *G. polynesiensis* is therefore thought to be an important contributor to CFP in the Pacific. Interestingly, a strain of *G. polynesiensis* was recently isolated and found not to produce CTXs when tested with LC-MS/MS, but this is highly unusual for this species [68]. Presently, only six strains of *G. polynesiensis* have been characterized in the literature, four from French Polynesia [23], one from the Cook Islands [39] and one from the Kermadec Islands [68]. These locations are all situated within the central South Pacific Ocean but CFP occurs across a much larger area (see review by Friedman et al. [3]), and CTXs are confirmed to be present in fish from many locations [69,70,71,72]. Therefore, either the distribution of *G. polynesiensis* is much larger than presently acknowledged, or there are other currently unidentified precursor toxins that contribute to CFP in other locations.

Four congeners of MTX have been described, MTX-1, MTX-2, MTX-3 and MTX-4 [30,73,74] and chemical detection methods exist for the two disulphated forms, MTX-1 and MTX-3. Using LC-MS/MS analyses, we did not detect MTX-1 in any of the strains isolated in this study. This structural form of MTX-1 was originally described from a strain of *Gambierdiscus* (FP) isolated from French Polynesia [73]. The species used for this original description is not known but MTX-1 has since only been identified in strains of *G. australes* from the Cook Islands [39], the Kermadec Islands [68,75] and Japan [74].

MTX-3 is a putative MTX first described by Holmes and Lewis [30]. It is structurally smaller than MTX-1 and MTX-2 but its complete structure, potency and mode of action remain unknown. Since the original description, MTX-3 has been found in all *Gambierdiscus* strains tested (T. Harwood pers comm), except for the temperate *G. carpenteri* strains isolated in this study [76]. The factors driving the differences in MTX-3 production between the tropical and temperate populations of *G. carpenteri* are unknown; however, our study shows that temperature is not one of them. MTX-4 is a recently described congener of MTX, found only to be present in strains of *G. excentricus* isolated from a variety of locations and is highly potent [74].

The Ca^2+^ influx SH-SY5Y cell FLIPR bioassay [38], showed that all strains of *Gambierdiscus* isolated from eastern Australia produce between two and four peaks displaying MTX-like activities. As LC-MS/MS analyses did not detect the presence of MTX-1 in these strains, the toxicity must therefore be attributed to other types of MTX. Pisapia et al. [74] tested a wide range of *Gambierdiscus* strains for the presence of the newly described toxin MTX-4 and found only *G. excentricus* produced this compound. It is therefore unlikely that the MTX-like activity in the strains tested in this study is related to the presence of MTX-4. Alternatively, MTX-2 was originally described from a strain of *Gambierdiscus* (NQ1) isolated from the central Great Barrier Reef, Australia [73]. As there is no chemical detection method for this congener of MTX, it is not known if any of the MTX-like activity peaks in this study are from MTX-2, however it is likely as the original description was based on strains originating from Australia.

MTXs are hydrophilic compounds and the likelihood of their contribution to human illness events was originally discarded due to their water solubility [72]. However, recent work has confirmed that MTXs can be retained in the viscera, liver and flesh of fish [77]. These recent findings, coupled with the consistent detection of MTX-like activity in all *Gambierdiscus* strains tested in this study and by others using functional assays (e.g., haemolytic activity [36,37], mouse bioassay [19,59] and the Ca^2+^ influx SH-SY5Y cell FLIPR bioassay [38]), as well as the extremely high potency of these toxins, represent compelling arguments toward the need for further investigations into the role of MTXs in CFP.

## 4. Materials and Methods

### 4.1. Sampling and Isolation

Epiphytes, including associated benthic microalgae, were removed from the surface of seagrass (*Zostera* sp.) collected from the Merimbula Inlet, New South Wales, Australia (36.8979° S, 149.9044° E) and macroalgae (*Padina* sp., *Laurencia* sp. and *Chnoospora* sp.) collected from the Heron Island lagoon, Queensland, Australia (23.4423° S, 151.9148° E) on 7 April 2014 (austral autumn) and 27 July 2014 (austral winter), respectively.

Single cells of *Gambierdiscus* spp. were isolated using the micropipette technique [78] and placed in individual wells of a 48-well clear microplate with 0.2 µm filtered sterilised and autoclaved seawater collected from each site and incubated in a plant growth chamber (Labec, Sydney, NSW, Australia) at 20 °C under ~100 µmol photons m^−2^ s^−1^ on a 12:12 light:dark cycle. Modified K medium [13] was gradually introduced as cells began to grow (i.e., 1:10 *v*/*v*, then increasing quantities until 1:1). Once isolates reached a concentration of approximately 20 cells per well, each was transferred to 25 cm^2^ (50 mL) sterile vented polystyrene tissue culture flasks (Falcon, Corning, NY, USA), oriented horizontally. Established cultures were then maintained in these vessels in modified K medium made from sterile oceanic seawater (salinity was approximately 32 ppt), under ~100 µmol photons m^−2^ s^−1^ on a 12:12 light:dark cycle.

### 4.2. Strain Identification

Cells from approximately 100 mL of each *Gambierdiscus* culture were harvested by centrifugation at 600× *g* for 10 min. DNA was extracted using a MoBio Soil DNA Extraction Kit following the manufacturer’s instructions and sent to a commercial service (Australian Genomic Research Facility (AGRF), Queensland, Australia) where the D1–D3 region of the large subunit (LSU) rDNA was amplified using the primers D1R-F [79] and D3-R [80] and the D8–D10 region amplified using the primers D8F and D10R [13]. These genetic markers were selected because they are commonly used for the *Gambierdiscus* genus and many sequences are publicly available for comparison. PCR amplifications were carried out in 50 µL reaction volumes containing AmpliTaq Gold 360 master mix, both forward and reverse primers (2.5 µm) and template at a concentration of 1 ng µL^−1^. Thermocycling conditions for the D1–D3 region were 95 °C for 5 min, 35 cycles at 95 °C for 30 s, 60 °C for 2 min, with a final step at 72 °C for 10 min. Thermocycling conditions for the D8–D10 region were 95 °C for 5 min, 35 cycles at 95 °C for 30 s, 54 °C for 30 s, 72 °C for 1 min, with a final step at 72 °C for 5 min. Amplification products (~950 bp) were purified and sequenced in both directions using the Sanger sequencing platform. 

Phylogenetic analyses were conducted in Genious v9.1.5 [81]. Publicly available sequences of *Gambierdiscus* spp. and sequences used as out-groups (*Akashiwo sanguinea, Prorocentrum micans* and *Alexandrium affine*, *A. catenella* and *A. tamarense*) were downloaded from GenBank (www.ncbi.nlm.nih.gov) and aligned with the sequences obtained from this study, using the MUSCLE algorithm (maximum number of iterations 8) [82]. Sequences from the D1–D3 and D8–D10 regions were truncated to 979 bp and 764 bp, respectively. Maximum Likelihood (ML) phylogenetic trees were generated for both regions with PHYML with 1000 bootstraps [83] using a GTR substitution model and an estimated gamma distribution. Bayesian analysis was performed for both regions using MrBayes 3.2.6 [84] by means of the GTR+G (general-time reversible with gamma-shaped among-site variation) model. Bayesian analyses were carried out in four simultaneous runs with four chains each for 3.1 × 10^6^ generations, sampling every 1000 trees and 1000 trees were discarded as burn in. 

### 4.3. Toxicology

#### 4.3.1. Detecting P-CTX-3B, P-CTX-3C, P-CTX-4A and P-CTX-4B Using Liquid Chromatography-Tandem Mass Spectrometry (LC-MS/MS)

To test for the presence of CTXs commonly linked with CFP (P-CTX-3B, P-CTX-3C, P-CTX-4A, P-CTX-4B), *Gambierdiscus* strains isolated in this study were cultured in triplicate 75 cm^2^ (250 mL) sterile vented polystyrene tissue culture flasks (Falcon, Corning, NY, USA), at 24 °C in a temperature-controlled room under the maintenance conditions described above. To test the effect of temperature on toxin production by the tropical and temperate strains of *G. carpenteri*, an experiment was performed where cultures of the tropical and temperate strains (UTSHI2C4 and UTSMER9A3, respectively) were grown at 18 or 27 °C in temperature-controlled plant growth cabinets (Climatron^®^, Plant Growth Cabinet, Australia).

Cell growth was monitored using in vivo chlorophyll *a* fluorescence measurements. A 1 mL aliquot was taken every 3 to 4 days from each flask and in vivo chlorophyll *a* fluorescence was measured for each sample using a fluorometer (Turner Designs, Trilogy^®^, San Jose, CA, USA) and then preserved with 1% Lugols iodine solution. Cultures were harvested in early stationary phase by centrifugation (3000× *g* for 10 min) and the three replicates were pooled to yield sufficient biomass before being freeze dried until further LC-MS/MS analyses. 

Lugols-preserved cells were used to estimate the number of cells in each cell pellet. The final 1 mL aliquot of culture collected prior to harvesting was counted using a Sedgewick Rafter Counting Chamber under an inverted light microscope (× 100 magnification) (Nikon Instruments, Nikon Eclipse TS100, Melville, NY, USA).

Analysis of selected CTXs was carried out using a quantitative LC-MS/MS method developed at the Cawthron Institute (full method details will be disclosed in an upcoming manuscript by T. Harwood). Microalgal pellets containing between 2.0 × 10^5^ and 2.0 × 10^6^ cells were extracted in 1 mL of 100% MeOH in glass tubes, then mixed and sonicated for 5 min. Samples were transferred to glass auto sampler vials using a glass pipette and 2 µL was injected for analysis. LC-MS/MS analysis was performed on an Ultra Performance Liquid Chromatography (UPLC) coupled to a mass spectrometer with electrospray ionization. Chromatographic separation used a BEH Phenyl column eluted with ammoniated mobile phases; (A) Milli-Q and (B) acetonitrile. Starting conditions were 25% B followed by a stepped gradient to 95% B after 8 min, with re-equilibration to 25% B between 8 and 9 min. A flow rate 0.55 mL min^−1^ was used and the total run time was 9 min. Microalgal CTX reference material (P-CTX-3B; P-CTX-3C; P-CTX-4A; P-CTX-4B) was supplied by Institut Louis Malardé, French Polynesia.

MTX-1 (limit of detection of 1 ng mL^−1^) and putative MTX-3 were monitored as intact structures using methods developed at Cawthron Institute [85,86]. Briefly, for MTX-1, a pseudo multiple reaction monitoring (MRM) transition (*m*/*z* 1689.6 > 1689.6) was acquired for the intact di-anion with the electrospray ionization source being operated in negative-ion mode. It was also possible to monitor the presence of the sulphated polyether analyte known as MTX-3 using a specific MRM transition (*m*/*z* 1037.6 > 96.8).

#### 4.3.2. Ciguatoxin (CTX) and Maitotoxin-Like (MTX) Activities

Selected strains of *Gambierdiscus* (tropical *G. carpenteri* UTSHI2C4, UTSHI6C3; temperate *G. carpenteri* UTSMER8B4, UTSMER9A3; *G. lapillus* UTSHI6B5, UTSHI2B6; *Gambierdiscus* sp. UTSHI6A6; *Gambierdiscus* sp. UTSHI6B1) were analysed for CTX and MTX-like activities. Strains were grown under standard conditions at 24 °C in a temperature-controlled room in 2 × 2 L glass Schott bottles. Chlorophyll *a* in vivo fluorescence was used as a proxy for cell abundance to track growth and duplicate bottles were pooled and harvested in the early stationary phase by first concentrating the cells on a 20 µm sieve, followed by centrifugation (3000× *g* for 10 min). Total cell abundances ranged between 1.0 × 10^6^ and 3.5 × 10^6^.

CTX and MTX-like activities were determined following the functional bioassay described by Lewis et al. [38]. Briefly, cell pellets were extracted twice in a mix of analytical grade methanol:ultrapure water:hexane (2:1:1) at a concentration of 10 mL per 1.0 × 10^6^ cells and subsequently sonicated twice for 1 min using a sonication probe (QSonica) at an amplitude of 50%. The extract was then centrifuged to remove cell debris (600× *g* for 10 min) and the supernatant layers sampled. The hexane layer was discarded and the remaining extract dried under N_2_ gas, then reconstituted in 10 mL dichloromethane (DCM) and extracted twice with 5 mL 60% methanol (MeOH). The DCM lipophilic phase containing CTXs (top layer) was then separated from the MeOH hydrophilic phase containing MTXs (bottom layer) and each phase was dried separately under N_2_ gas in 20 mL amber glass vials.

The dried extracts were reconstituted in 30% acetonitrile (ACN)/0.1% formic acid (FA) and approximately 1.0 × 10^6^ microalgal cell equivalents were used for fractionation. Extracts were fractionated on an UltiMate 3000 Rapid Separation Liquid Chromatography System (Dionex, IL, USA) with a FC 204 Fraction Collector (Gilson, Middleton, WI, USA). Grace Vydac C18 (218TP) Reverse-Phase HPLC Column (250 × 4.6 mm, 5 µm) (Grace Hichrom, Berkshire, UK) was used to separate the extracts with 0.043% trifluoroacetic acid/90% acetonitrile (aq) as elution buffer B and 0.05% trifluoroacetic acid (aq) as buffer A. Initial elution was at 5% B for 5 min and then increased linearly to 90% B over 60 min at a flow rate of 0.7 mL min^−1^. 77 fractions were collected, freeze dried and reconstituted in 30 µL physiological salt solution (PSS; composition NaCl 140 mM, glucose 11.5 mM, KCl 5.9 mM, MgCl_2_ 1.4 mM, NaH_2_PO_4_ 1.2 mM, NaHCO_3_ 5 mM, CaCl2 1.8 mM, HEPES 10 mM) containing 0.1% bovine serum albumin (BSA) just prior to Ca^2+^ influx SH-SY5Y cell FLIPR bioassay analysis.

Functional activity of extracts was then determined following Lewis et al. [38]. Briefly, SH-SY5Y human neuronal cells (ECACC, Salisbury, Wiltshire, UK) were maintained in Roswell Park Memorial Institute (RPMI) media containing 15% Foetal Bovine Serum (FBS) and 2 mM l-glutamine, 100 units mL^−1^ penicillin and 0.1 mg L^−1^ streptomycin at 37 °C, under 5% CO_2_ atmosphere. Cells were routinely split once a week at a 1:5 dilution using 0.25% trypsin with ethylenediaminetetraacetic acid (EDTA) (Gibco).

For the Ca^2+^ influx FLIPR bioassay, SH-SY5Y cells were planted into 384-well black walled imaging plates (Corning, Australia) at a density of 50,000 cells per well and cultured for 48 h. Fluorescent responses (excitation, 470–495 nm; emission, 515–575 nm) were assessed using the FLIPR^TETRA^ Fluorescent Imaging Plate Reader (Molecular Devices, Sunnydale, CA, USA) after a 30 min incubation with a fluorescent Ca^2+^ dye (Calcium 4 No Wash Dye, Molecular Devices) diluted in PSS containing 0.1% BSA. Ten microliters of reconstituted HPLC fractions were added to each well and the fluorescence response recorded for 5 min prior to stimulation with 5 µM veratridine. Signals were interpreted as follows: If MTX-like activity was detected in the sample, calcium influx would be evident in this early stage of the assay. If a second peak was detected in response to veratridine addition, this was interpreted as an effect of CTX-like activity which acts to enhance an otherwise sub-effective dose of veratridine. Positive control standards of P-CTX-2 and P-CTX-3 were used to verify CTX-like activity. The assay detected P-CTX-2 at 9.51 ± 0.13 and P-CTX-3 at 9.26 ± 0.14, (pEC_50_, *n* = 2) in the presence of 5 µm veratridine, consistent with previous results using this assay by Lewis et al. [38].

FLIPR assay data were analysed using ScreenWorks 3.2.0.1.4 (Molecular Devices, Sunnydale, CA, USA). For each fraction, the MTX-like activity was normalized to the baseline and the maximum peak height in the following 300 reads represented the MTX-like response. CTX-like activity was normalized to the veratridine response and the maximum peak height after the veratridine addition represented the CTX-like response.

## 5. Conclusions

In this study, 14 strains of *Gambierdiscus* were established from eastern Australia, representing four species. Extracts of three species showed CTX-like activity, likely caused by compounds which are distinct from currently characterized toxins. These results suggest that toxins produced by these Australian strains of *Gambierdiscus* likely differ from those currently linked with CFP identified in extracts of *G. polynesiensis* from French Polynesia, though characterization of the compounds responsible for the CTX-like activity is necessary to evaluate this. Although this study was limited to Australian strains of *Gambierdiscus*, the findings are likely universal and therefore, a concentrated effort should be made toward testing and characterizing the toxicology of strains from other locations. Future research should also include establishing a baseline of *Gambierdiscus* species distribution in Australia, understanding the relative abundance of each species and finding out how these organisms are influenced by environmental conditions, particularly in view of a changing climate. 

## Figures and Tables

**Table 1:** Toxicology of *Gambierdiscus* species documented from the Australian region. NA denotes information is not available.

Species	Location	Toxicity	LC-MS/MS Profile	Reference
*G.* cf. *belizeanus*	Heron Island, Queensland	NA	NA	[55]
*G. carpenteri*	Townsville, Queensland	NA	NA	[55,56]
	Merimbula and Wapengo Lagoon, New South Wales	NA	No CTX-3B, -3C, -4A, -4B No MTX-1, or MTX-3	[57]
*G. honu*	Heron Island, Queensland	NA	NA	[15,40]
*G. lapillus*	Heron Island, Queensland	NA	No CTX-3B, -3C, -4A, -4B No MTX-1 MTX-3 present	[58]
*G. toxicus* *	Arlington Reef, Queensland Platypus Bay, Queensland	Positive	NA	[49]

* The identification of *G. toxicus* is not certain as the study occurred prior to revision of the genus.

**Table 2:** Geographic origin and toxicity of *Gambierdiscus* strains established in this study. All strains were grown at 24 °C for toxin analysis. Strains listed with temperatures (e.g., 18 °C and 27 °C), indicate additional growth temperatures that were tested. For the LC-MS/MS analysis, ND denotes toxins not detected. For the Ca ^2+^ influx SH-SY5Y cell FLIPR bioassay, results are shown as the number of CTX- and MTX-like peaks of activity and NA signifies strains which were not analysed.

Species Name	Site of Isolation	Strain Number	Genbank Accession No.		LC-MS/MS		Ca^2+ ^Influx SH-SY5Y Cell FLIPR Bioassay	
			LSU D1–D3	LSU D8–D10	CTX	MTX	CTX-Like Activity	MTX-Like Activity
Tropical G. carpenteri	Heron Island Lagoon, Australia	UTSHI2C4	MG280880	MG280866	ND	MTX-3	0	3
Tropical G. carpenteri (18 °C)					ND	MTX-3	NA	NA
Tropical G. carpenteri (27 °C)					ND	MTX-3	NA	NA
Tropical G. carpenteri	Heron Island Lagoon, Australia	UTSHI6C3	MG280881	MG280869	ND	MTX-3	0	4
Tropical G. carpenteri	Heron Island Lagoon, Australia	UTSHI6A1	MG280883	MG280872	ND	MTX-3	NA	NA
Tropical G. carpenteri	Heron Island Lagoon, Australia	UTSHI6D2	MG280884	MG280873	ND	MTX-3	NA	NA
Temperate G. carpenteri	Merimbula Inlet, Australia	UTSMER9A3	MG280889	MG280878	ND	ND	0	4
Temperate G. carpenteri (18 °C)					ND	ND	NA	NA
Temperate G. carpenteri (27 °C)					ND	ND	NA	NA
Temperate G. carpenteri	Merimbula Inlet, Australia	UTSMER8B4	MG280886	MG280875	ND	ND	0	4
Temperate G. carpenteri	Merimbula Inlet, Australia	UTSMER7A1	MG280887	MG280876	ND	ND	NA	NA
Temperate G. carpenteri	Merimbula Inlet, Australia	UTSMER1A3	MG280888	MG280877	ND	ND	NA	NA
Temperate G. carpenteri	Merimbula Inlet, Australia	UTSMER8A4	MG280890	MG280879	ND	ND	NA	NA
G. lapillus	Heron Island Lagoon, Australia	UTSHI6B5		MG280867	ND	MTX-3	1	3
G. lapillus	Heron Island Lagoon, Australia	UTSHI2B6		MG280868	ND	MTX-3	1	2
G. lapillus	Heron Island Lagoon, Australia	UTSHI2B5		MG280871	ND	MTX-3	NA	NA
Gambierdiscus sp.	Heron Island Lagoon, Australia	UTSHI6A6	MG280882	MG280870	ND	MTX-3	1	2
Gambierdiscus sp.	Heron Island Lagoon, Australia	UTSHI6B1	MG280885	MG280874	ND	MTX-3	1	3
